# Dynamic Surface Activity of a Fully Synthetic Phospholipase-Resistant Lipid/Peptide Lung Surfactant

**DOI:** 10.1371/journal.pone.0001039

**Published:** 2007-10-17

**Authors:** Frans J. Walther, Alan J. Waring, Jose M. Hernandez-Juviel, Larry M. Gordon, Adrian L. Schwan, Chun-Ling Jung, Yusuo Chang, Zhengdong Wang, Robert H. Notter

**Affiliations:** 1 Los Angeles Biomedical Research Institute, Harbor-University of California at Los Angeles (UCLA) Medical Center, Torrance, California, United States of America; 2 Department of Pediatrics, Leiden University Medical Center, Leiden, The Netherlands; 3 Department of Medicine, University of California at Los Angeles, Los Angeles, California, United States of America; 4 Department of Chemistry, University of Guelph, Guelph, Ontario, Canada; 5 Department of Pediatrics, University of Rochester, Rochester, New York, United States of America; 6 Department of Environmental Medicine, University of Rochester, Rochester, New York, United States of America; Stanford University, United States of America

## Abstract

**Background:**

This study examines the surface activity and resistance to phospholipase degradation of a fully-synthetic lung surfactant containing a novel diether phosphonolipid (DEPN-8) plus a 34 amino acid peptide (Mini-B) related to native surfactant protein (SP)-B. Activity studies used adsorption, pulsating bubble, and captive bubble methods to assess a range of surface behaviors, supplemented by molecular studies using Fourier transform infrared (FTIR) spectroscopy, circular dichroism (CD), and plasmon resonance. Calf lung surfactant extract (CLSE) was used as a positive control.

**Results:**

DEPN-8+1.5% (by wt.) Mini-B was fully resistant to degradation by phospholipase A_2_ (PLA_2_) *in vitro,* while CLSE was severely degraded by this enzyme. Mini-B interacted with DEPN-8 at the molecular level based on FTIR spectroscopy, and had significant plasmon resonance binding affinity for DEPN-8. DEPN-8+1.5% Mini-B had greatly increased adsorption compared to DEPN-8 alone, but did not fully equal the very high adsorption of CLSE. In pulsating bubble studies at a low phospholipid concentration of 0.5 mg/ml, DEPN-8+1.5% Mini-B and CLSE both reached minimum surface tensions <1 mN/m after 10 min of cycling. DEPN-8 (2.5 mg/ml)+1.5% Mini-B and CLSE (2.5 mg/ml) also reached minimum surface tensions <1 mN/m at 10 min of pulsation in the presence of serum albumin (3 mg/ml) on the pulsating bubble. In captive bubble studies, DEPN-8+1.5% Mini-B and CLSE both generated minimum surface tensions <1 mN/m on 10 successive cycles of compression/expansion at quasi-static and dynamic rates.

**Conclusions:**

These results show that DEPN-8 and 1.5% Mini-B form an interactive binary molecular mixture with very high surface activity and the ability to resist degradation by phospholipases in inflammatory lung injury. These characteristics are promising for the development of related fully-synthetic lipid/peptide exogenous surfactants for treating diseases of surfactant deficiency or dysfunction.

## Introduction

Endogenous pulmonary surfactant contains a complex mix of ester-linked glycerophospholipids and specific apoproteins that interact biophysically to produce the surface properties needed for functional activity at the alveolar interface [1]. Current exogenous surfactant drugs used to treat lung disease or injury in pediatric and adult patients also contain a substantial content of ester-linked glycerophospholipids including dipalmitoyl phosphatidylcholine (DPPC). The surface activity of endogenous or exogenous surfactants becomes compromised if DPPC or other essential glycerophospholipids are chemically degraded or structurally altered in the alveoli. One important cause of such effects is through the action of phospholipases in the lungs during inflammatory injury [2]–[9]. Phospholipase-induced degradation of lung surfactant glycerophospholipids not only reduces the concentration of active components, but also generates reaction products such as lysophosphatidylcholine and fluid free fatty acids that can further decrease surface activity by interacting biophysically with remaining surfactant at the alveolar interface [10]–[12].

Synthetic exogenous surfactants containing novel lipids resistant to degradation by phospholipases have the potential to maintain high activity when these lytic enzymes are present in the inflammatory response during clinical acute lung injury (ALI) and the acute respiratory distress syndrome (ARDS) [13]–[17]. The incidence of ALI has been estimated as 20–65 cases per 100,000 persons per year in the United States, with approximately 50–150,000 adults developing ARDS (all patients with ARDS also by definition have ALI) [18], [19]. Surfactant dysfunction from physical or chemical interactions with endogenous inhibitors during acute pulmonary injury has been extensively documented (e.g., [1], [20], [21] for review). Although the pathophysiology of ALI/ARDS is complex and includes inflammation, vascular dysfunction and cell/tissue injury in addition to surfactant dysfunction, the latter is an important contributor to respiratory failure in many patients and provides a rationale for therapy with exogenous surfactants.

Synthetic exogenous surfactant preparations have significant potential advantages as pharmacologic products compared to animal-derived clinical surfactants, including improved compositional and activity reproducibility, easier and less-costly quality control, freedom from prions or other biologic agents, and reduced ethnographic (cultural/religious) concerns relating to animal species. This paper investigates the surface activity of a novel fully-synthetic exogenous surfactant that contains DEPN-8, a phospholipase-resistant C16:0 diether phosphonolipid analog of DPPC reported previously by Notter, Schwan, Turcotte, and co-workers [22]–[24]. The synthetic surfactant studied also contains Mini-B, a 34 amino acid peptide designed to retain major amphipathic regions of highly-active human surfactant protein (SP)-B [25]. The molecular interactions of Mini-B and DEPN-8 are defined here by Fourier transform infrared (FTIR) spectroscopy, circular dichroism (CD) and plasmon resonance binding affinity, and the surface activity of DEPN-8+1.5% Mini-B is assessed in adsorption experiments and by measurements on both the pulsating and captive bubble surfactometers. These two bubble surfactometers are specifically designed to define the overall surface tension lowering activity of lung surfactant dispersions in physical systems that incorporate a range of relevant surface behaviors including dynamic film compression, spreading, and adsorption to the air-water interface [1]. Comparative surface studies investigate calf lung surfactant extract (CLSE), which has documented high activity in reversing states of surfactant deficiency in mammalian lungs, and is the substance of the clinical surfactant Infasurf® [1], [21], [26].

## Results

### Circular dichroism (CD) and FTIR spectroscopy on Mini-B in TFE or DEPN-8

**Figure 1:**
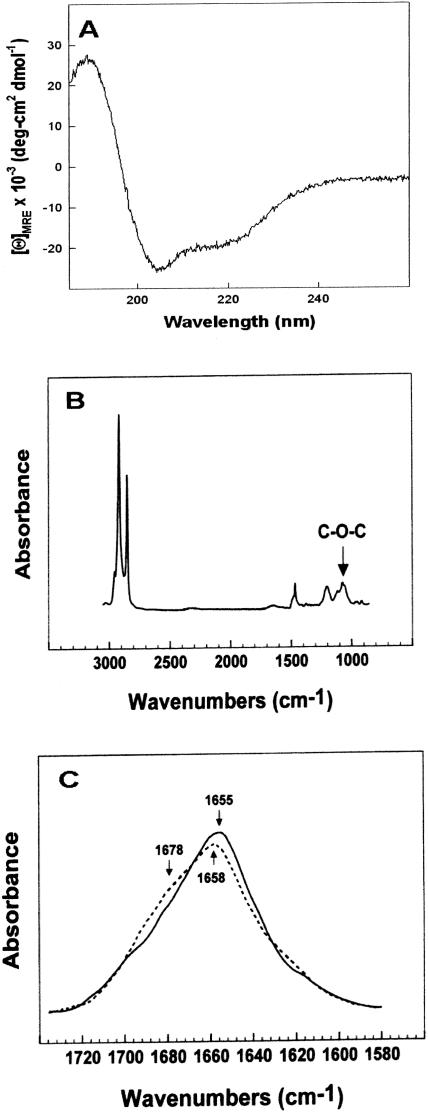
Spectroscopic behavior of Mini-B and DEPN-8. Panel A: CD spectrum for Mini-B in trifluoroethanol (TFE); Panel B: FTIR spectrum for DEPN-8; Panel C: FTIR spectral differences for Mini-B in DEPN-8 (dashed line) compared to Mini-B in TFE (solid line). In Panel A, mean residue ellipticity (MRE) averaged over eight scans is plotted against wavelength for Mini-B in 4∶6 (v:v) TFE:10 mM phosphate buffer, pH 7.4. The double minimum at ∼208 and 222 nm is indicative of a high α-helical content. In Panel B, the spectrum for DEPN-8 multilayers (100 µg lipid, arbitrary absorbance units) has a “C-O-C” ether linkage-associated absorption band centered at a wavenumber of 1072 cm^−1^. In Panel C, the IR spectrum of Mini-B in TFE (solid line) has a peak at 1655 cm^−1^ indicating high α-helix levels, while the peak at 1658 cm^−1^ and high-field shoulder at 1678 cm^−1^ for Mini-B in DEPN-8 (dashed line) indicates an increase in turn/bend conformation with a decreased but still prominent α-helix content. See text for discussion.

CD spectroscopy was used to examine the conformation of Mini-B in phosphate buffered trifluoroethanol (TFE, pH = 7.4), a solvent environment that partially mimics the polar/amphipathic region near the aqueous interface of a bilayer membrane [27]. A representative CD spectrum for Mini-B in TFE in the wavelength region between 185 and 260 nm is shown in Figure 1A. The spectrum shows a double minimum at approximately 208 nm and 222 nm, consistent with a substantial α-helical content. Analysis of the CD spectrum by the methods of Sreerama et al [28] indicated mean percent conformations of about 41.4% α-helix, 22% turn/bend, 14.3% β-sheet, and 22.3% disordered structures (Table 1). Additional CD spectra for Mini-B in multilayers of DEPN-8 in phosphate buffered saline exhibited low signal/noise ratios due to excessive light-scattering (data not shown), and were not analyzed for conformation. Instead, Mini-B in the presence of DEPN-8 was studied using FTIR spectroscopy, which is not subject to light-scattering artifacts. FTIR was also used to assess DEPN-8 in the absence of Mini-B.

**Table 1:** Mean proportions of different aspects of secondary structure for Mini-B in structure-promoting TFE solvent or in deuterium-hydrated DEPN-8 multilayers based on CD and FTIR spectroscopic analysis.

Sample *	% Conformation			disordered
	α-helix	turn/bend	β-sheet	
Mini-B in TFE (CD)	41.4	22.0	14.3	22.3
Mini-B in TFE (FTIR)	37.1	33.5	10.6	17.8
Mini-B in DEPN-8 (FTIR)	27.2	43.6	10.5	18.7

* CD spectra for Mini-B in TFE were analyzed for secondary structure using the methods of Sreerama et al [28], and FTIR spectra were analyzed for secondary conformation based on deconvolution of the amide I band (Methods). FTIR spectra for Mini-B in deuterium-hydrated DEPN-8 multilayers were done at a molar ratio of 10∶1 lipid∶peptide. Tabulated results are means from four closely-reproduced separate determinations for each condition and spectral type.

Multilayers of DEPN-8 exhibited a strong C-O-C absorption band between wavenumbers of 1004–1157 cm^−1 ^(peaking at ∼1072 cm^−1^, Figure 1B), consistent with behavior previously shown for phospholipids with ether-linked alkyl chains [29]. DEPN-8 multilayers also had an absorbance peak at wavenumbers of ∼1220 to 1260 cm^−1^ indicative of the asymmetric stretching frequencies of the polar headgroup, as well as contributions from -CH_2_- scissoring absorption in the spectral region of 1462 to 1473 cm^−1^. Dominant absorptions for the alkyl chains that included antisymmetric and symmetric stretching bands around 2917 and 2850 cm^−1^ were also found (Figure 1B). DEPN-8 multilayers did not exhibit absorption in the region of 1710–1740 cm^−1^, which is characteristically associated with the C = O stretch of normal ester linkages in glycerophospholipids.

FTIR spectra for Mini-B in TFE and in DEPN-8 were similar, with substantial overlapping regions but some small variations (Figure 1C). The FTIR spectrum of Mini-B in TFE had a major amide I band centered at 1655 cm^−1^, indicating a predominant α-helical conformation. FTIR spectral deconvolution analysis indicated mean secondary structure percentages for Mini-B in TFE of 37.1% α-helix, 33.5% turn/bend, 10.6% β-sheet, and 17.8% disordered. This structural distribution is similar to that obtained from the CD spectrum of Mini-B in TFE (Table 1), with the largest difference being a higher percentage of turn/bend structures in the FTIR analysis compared to the CD analysis. Deconvolution of the FTIR spectrum of Mini-B in DEPN-8 indicated a further increase in the proportion of turn/bend elements relative to the FTIR spectrum of Mini-B in TFE (an increase in turn/bend structures to 43.6% indicated by a minor shoulder centered at ∼1678 cm^−1^, with a decrease in α-helix to 27.2% indicated by a peak shift to 1658 cm^−1^) (Figure 1C; Table 1). These FTIR results indicate direct interactions between Mini-B and DEPN-8 at the molecular level.

### Biacore plasmon resonance binding affinities of Mini-B for lipids

Molecular binding affinities (associations) between chip-linked films of Mini-B peptide and liposomes of DEPN-8 or DPPC were measured at 37°C using a Biacore apparatus. Results showed that Mini-B had a substantial binding (association) affinity for both DEPN-8 and DPPC based on a high uptake rate constant (k_on_) and a low dissociation rate constant (k_off_) (Table 2). DEPN-8 had a slightly higher k_on_ rate for Mini-B compared to DPPC, but the diether lipid also had a higher mean k_off_ rate. Values for the mean equilibrium dissociation constant (KD = k_off_/k_on_) were low and similar for DEPN-8 (104 nM) and DPPC (89 nM), showing that both lipids had substantial molecular affinity for the chip-linked Mini-B monolayer (Table 2).

**Table 2:** Mean association and dissociation kinetic rate constants (k_on_, k_off_) and equilibrium dissociation constant KD calculated from plasmon resonance measurements for liposomes of DEPN-8 or DPPC flowing past a chip-linked Mini-B monolayer.

Lipid compound*	k_on_ (1/Ms)	k_off_ (1/s)	KD (nM)
DEPN-8	12.5×10^4^	13×10^−3^	104
DPPC	11.9×10^4^	9.9×10^−3^	83.2

* Liposomes of DEPN-8 or DPPC in running buffer (10 mM HEPES, 150 mM NaCl, 3 mM EDTA, 0.005% Surfactant P20, pH 7.4) were flowed past a monolayer of Mini-B linked via Cys4 and Cys27 to a C5M sensor chip in a Biacore 3000 system (Methods). Mean kinetic rate constants (k_on_, k_off_) and the equilibrium dissociation constant (KD = k_off_/k_on_) were determined from curve fitting analyses of plasmon resonance results at six different lipid concentrations (0.1, 0.2, 0.3, 0.4, 0.5, and 0.6 µg/ml for each lipid).

### Resistance of synthetic surfactants containing DEPN-8+1.5% by weight Mini-B to degradation by phospholipase A_2_ (PLA_2_)

The structural resistance of DEPN-8 to degradation by phospholipases is a potential advantage for this compound as a constituent in novel exogenous surfactants for use in inflammatory lung injuries where lytic enzymes of this kind are released. Mixtures of DEPN-8+1.5% Mini-B were incubated *in vitro* with 0.1 Units of PLA_2_, and completely resisted degradation from this enzyme based on thin layer chromatographic analysis (Table 3). In contrast, CLSE is significantly degraded by PLA_2_, with a substantial decrease in its content of phosphatidylcholine and a substantial increase in lysophosphatidylcholine as reported in our prior work [30].

**Table 3:** Resistance of DEPN-8+1.5% by weight Mini-B to degradation by phospholipase A_2_ (PLA_2_) compared to calf lung surfactant extract (CLSE).

Lipid Class	CLSE	CLSE+PLA_2_	DEPN-8+1.5% Mini-B	DEPN-8+1.5% Mini-B+PLA_2_
Lysophosphatidylcholine	0.4±0.2	29.5±2.4		
Sphingomyelin	1.0±0.2	1.2±0.5		
Phosphatidylcholine	84.4±0.4	55.1±3.2	100	100
Phosphatidylinositol	4.0±0.6	3.8±0.7		
Phosphatidylethanolamine	3.7±0.7	3.8±1.0		
Phosphatidylglycerol	4.7±0.3	4.1±0.6		
Residue	1.8±0.2	2.5±0.2		

Data are mean±SEM for n = 3. DEPN-8+1.5% by weight Mini-B was incubated *in vitro* with PLA_2_ (0.1 Units/ml) for 30 min at 37°C, and degradation was assessed by measuring lipid classes in weight percent based on phosphate analysis of bands on thin layer chromatography. Results for CLSE in the presence and absence of PLA_2_ utilized identical methods as reported previously by Wang et al [30].

### Adsorption and pulsating bubble surface activity of synthetic lung surfactants containing DEPN-8+1.5% by weight Mini-B peptide

**Figure 2:**
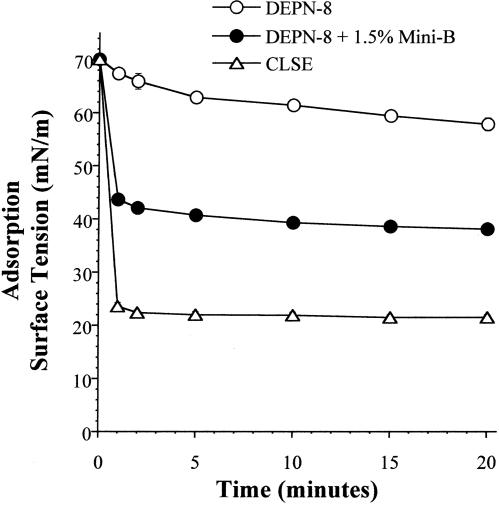
Adsorption of DEPN-8 with and without 1.5% (by wt) Mini-B compared to calf lung surfactant extract (CLSE). Adsorption surface tensions are plotted following the addition of a bolus of DEPN-8, DEPN-8+1.5% Mini-B, or CLSE to a stirred subphase (10 mM HEPES with 0.15M NaCl and 1.5 mM CaCl_2_ at pH 7.0) in a Teflon® dish at time zero. Final subphase surfactant concentration was uniform at 0.0625 mg lipid/ml. Data are Mean±SEM for n = 3–5. See text for details.

**Figure 3:**
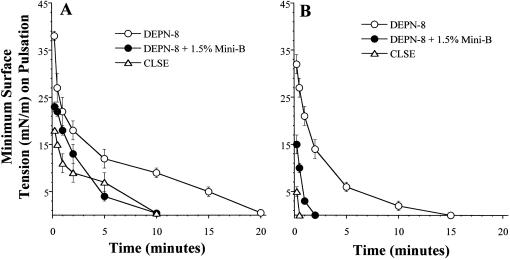
Dynamic surface activity of DEPN-8+1.5% (by wt) Mini-B compared to CLSE on the pulsating bubble surfactometer. Panel A: 0.5 mg/ml phosphonolipid (phospholipid); Panel B: 2.5 mg/ml phosphonolipid (phospholipid). Surface tension at minimum bubble radius (minimum surface tension) for DEPN-8+1.5% Mini-B and CLSE is graphed as a function of time on a pulsating bubble surfactometer (37°C, 20 cycles/min, 50% area compression). Data are Mean±SEM for n = 3–5. See text for details.

Combining Mini-B with DEPN-8 in a binary mixture significantly improved adsorption to the air-water interface (Figure 2). DEPN-8 alone reached adsorption surface tensions of 67.4±0.6 mN/m (at 1 min) and 57.8±1.2 mN/m (at 20 min) when injected into a stirred subphase. In contrast, DEPN-8+1.5% Mini-B reached much lower surface tensions of 43.7±0.8 mN/m and 38.1±0.7 mN/m after 1 and 20 min of adsorption, respectively. The greatest adsorption was exhibited by CLSE, which reached surface tensions of 23.6±0.7 mN/m at 1 min and 21.5±0.5 mN/m at 20 min following injection into the subphase (Figure 2). DEPN-8+1.5% Mini-B and CLSE both exhibited very high dynamic surface activity in studies on the pulsating bubble surfactometer (Figure 3). At a low phosphonolipid concentration of 0.5 mg/ml, DEPN-8+1.5% Mini-B reached minimum surface tensions of 4±1 mN/m (at 5 min of pulsation) and <1 mN/m (at 10 min of pulsation) (Figure 3A). CLSE (0.5 mg/ml) had equivalent minimum surface tension values of 7±2 mN/m and <1 mN/m at these times of bubble pulsation (Figure 3A). When surfactant concentration was raised to 2.5 mg/ml, dynamic surface activity was increased for all surfactants (Figure 3B). At 2.5 mg/ml, DEPN-8+1.5% Mini-B and CLSE reached minimum surface tensions of <1 mN/m by 2 min and 0.5 min of bubble pulsation, respectively. In comparison, DEPN-8 alone at 2.5 mg/ml had a minimum surface tension of 14±2 mN/m at 2 min, and required 15 min of bubble pulsation to reach values of <1 mN/m (Figure 3B). Although minimum surface tension is a primary indicator of lung surfactant activity, maximum surface tensions were also assessed in pulsating bubble studies. Maximum surface tension values for DEPN-8+1.5% Mini-B during cycling on the pulsating bubble apparatus were 9–20 mN/m higher than those of CLSE at a given surfactant concentration (data not shown). Detailed values of maximum surface tension during cycling for DEPN-8+1.5% Mini-B and CLSE are shown later for studies on the captive bubble surfactometer.

### Ability of DEPN-8+1.5% Mini-B and CLSE to reach minimum surface tensions <1 mN/m in the presence of serum albumin on the pulsating bubble

**Figure 4 pone-0001039-g004:**
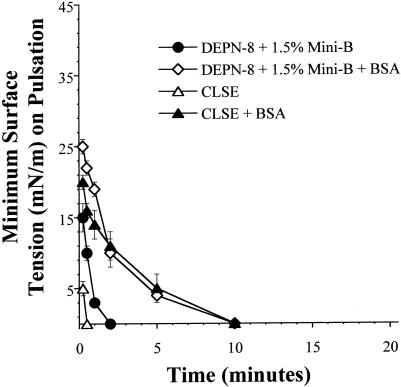
Surface activity of DEPN-8+1.5% (by wt) Mini-B and CLSE in the presence of bovine serum albumin. Surface tension at minimum radius (minimum surface tension) is graphed as a function of time for DEPN-8+1.5% Mini-B and CLSE in the presence of bovine serum albumin (3 mg/ml) on a pulsating bubble surfactometer (37°C, 20 cycles/min, 50% area compression). Surfactant concentration was 2.5 mg/ml of phosphonolipid (phospholipid). Data are Mean±SEM for n = 4–5.

Albumin is an important endogenous plasma protein known to biophysically inhibit the activity of endogenous and exogenous lung surfactants (e.g., [11], [21], [31]). At a surfactant phospholipid concentration of 2.5 mg/ml, both DEPN-8+1.5% Mini-B and CLSE had a prolonged timescale of surface tension lowering in the presence of serum albumin (3 mg/ml) (Figure 4). However, the overall activity curves for the two surfactant preparations were very similar, with both reaching minimum surface tensions of <1 mN/m by 10 min of bubble pulsation (Figure 4). The ability of synthetic DEPN-8+1.5% Mini-B to exhibit comparable surface tension lowering to CLSE in the presence of 3 mg/ml albumin is a positive finding, since prior work has established that CLSE is more resistant to this plasma protein than several other current clinical exogenous surfactants [1], [32]–[34].

### Surface-active behavior of DEPN-8+1.5% or 3% by weight Mini-B on the captive bubble surfactometer

**Figure 5 pone-0001039-g005:**
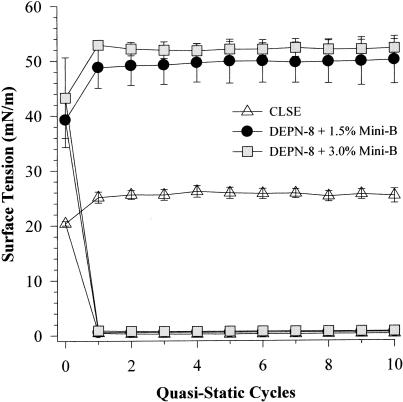
Quasi-static surface activity of DEPN-8+1.5% or 3% Mini-B compared to CLSE on the captive bubble surfactometer. Minimum and maximum surface tensions are shown for DEPN-8+1.5% or 3% by weight Mini-B compared to CLSE on a captive bubble surfactometer during slow compression (10 cycles over 90 min including a 2 min pause between each cycle). Surface tension values are Mean±SEM for at least three separate experiments. See text for details.

**Figure 6:**
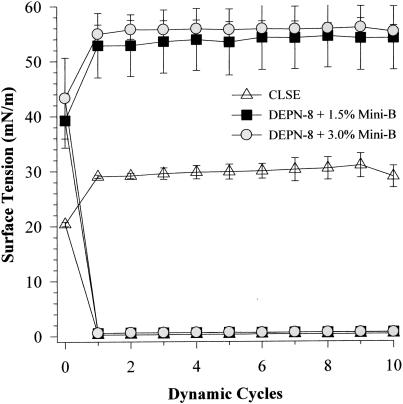
Dynamic surface activity of DEPN-8+1.5% or 3% Mini-B compared to CLSE on the captive bubble surfactometer. Minimum and maximum surface tensions are shown for DEPN-8+1.5% or 3% by weight Mini-B and CLSE on a captive bubble surfactometer during 10 cycles of rapid compression (20 cycles/min) following slow compression as in Figure 5. Surface tension values are Mean±SEM for at least three separate experiments. See text for details.

The interfacial behavior of DEPN-8+1.5% or 3% Mini-B is shown during 10 successive cycles of compression/expansion on the captive bubble surfactometer in Figures 5 and 6. Both of these synthetic mixtures were equivalent to CLSE in reaching minimum surface tensions of <1 mN/m on all ten recorded cycles of captive bubble compression/expansion at either a quasi-static rate (Figure 5) or at a dynamic rate (Figure 6). However, maximum surface tension values for DEPN-8+1.5% or 3.0% Mini-B for all cycles were greater than those of CLSE at the quasi-static and dynamic compression rates studied on the captive bubble (Figures 5 and 6, respectively).

## Discussion

The results of this study show that a binary synthetic lung surfactant containing DEPN-8+1.5% by weight Mini-B peptide had substantial surface activity that in several aspects approached the clinically-relevant bovine surfactant extract CLSE. Moreover, DEPN-8+1.5% Mini-B was fully resistant to chemical degradation when incubated *in vitro* with PLA_2_ (Table 2), while CLSE was severely degraded by this enzyme [30]. Mini-B and DEPN-8 had direct intermolecular interactions based on plasmon resonance binding affinity (Table 2) and on deconvolution analyses of FTIR spectra indicating a modified peptide secondary structure in multilayers with DEPN-8 (Fig. 1, Table 1). The adsorption of DEPN-8+1.5% Mini-B was greatly increased compared to DEPN-8 alone, although adsorption of the binary synthetic surfactant was less than that of CLSE (Fig. 2). DEPN-8+1.5% Mini-B had overall dynamic surface tension lowering ability in pulsating bubble studies that was similar to CLSE at a low surfactant phospholipid concentration of 0.5 mg/ml, with both surfactants reaching minimum surface tensions of <1 mN/m after 10 min of cycling (Fig. 3A). DEPN-8+1.5% Mini-B and CLSE also had comparable activity in reaching minimum surface tensions <1 mN/m in the presence of serum albumin at a surfactant concentration of 2.5 mg/ml on the pulsating bubble (Fig. 4). Complementary captive bubble studies showed that DEPN-8+1.5% or 3% Mini-B and CLSE all reached minimum surface tensions <1 mN/m when compressed under either quasi-static (Fig. 5) or dynamic (Fig. 6) conditions. However, maximum surface tension values for DEPN-8+1.5% Mini-B were higher than for CLSE on both the pulsating and captive bubble surfactometers. The sum of these findings show that DEPN-8 and Mini-B form an interactive and highly surface-active binary mixture, and support the continued development of related fully-synthetic exogenous lung surfactants containing novel lipids and SP-B peptides.

We have previously reported the high surface activity and inhibition resistance of model surfactants containing DEPN-8 or a C16:0 sulfur-containing ether analog (SO_2_-lipid) combined with 1.5% by weight of column-isolated bovine SP-B/C [30], [35]–[37]. These prior studies with purified native SP-B/C provide a proof of concept for the current work using Mini-B in a fully-synthetic binary lipid/peptide surfactant with DEPN-8. Mixtures of DEPN-8 or SO_2_-lipid+1.5% bovine SP-B/C rapidly reduce surface tension to <1 mN/m in the presence of albumin or C18:1 lysophosphatidylcholine (LPC) [30], [35]–[37]. DEPN-8+1.5% bovine SP-B/C has surface activity equal to CLSE when exposed to albumin, and surface activity superior to CLSE when exposed to PLA_2_ or LPC [30], [37]. The ability of DEPN-8+1.5% bovine SP-B/C to resist inhibition by PLA_2_, albumin or LPC to an equal or greater extent than CLSE in these prior studies is impressive, since this calf lung surfactant extract is known to have high activity in mitigating surfactant deficiency and/or dysfunction in animal models and patients ([1], [20], [38] for review). Results here showed that DEPN-8+1.5% Mini-B also had similar surface activity to CLSE in the presence of albumin (Fig. 4), indicating that related inhibition resistance characteristics can be achieved by a fully-synthetic lung surfactant. Further studies extending these findings to include inhibitors like LPC and also investigating other lipid/peptide synthetic surfactants will be important for future work.

In developing optimal fully-synthetic lung surfactants, it is challenging to substitute for the highly active full-length native surfactant proteins, which have strong molecular interactions with phospholipids. Among the surfactant apoproteins, SP-B is known to be particularly active in improving the adsorption and film behavior of lipids [1], [39]–[47]. The Mini-B used here was designed to maintain several important structural features of full-length human SP-B [25]. The N- and C-terminal domains of full-length SP-B are active sites of interaction with surfactant lipids [48]–[51], and Mini-B incorporates residues 8–25 and 63–78 of human SP-B that contribute to these amphipathic helices. Critical N- and C-terminal regions are joined in Mini-B via a β-sheet/loop domain. Peptide folding during synthesis is facilitated by specific solvents to produce the requisite helix hairpin structure stabilized by oxidation of cysteine residues, allowing Mini-B to form disulfide connectivities between Cys-8 and Cys-78 and Cys-11 and Cys-71 analogous to those in native SP-B (residue numbers refer to the full-length sequence of human SP-B) [25]. FTIR analyses and plasmon resonance binding affinity studies here confirmed that the structure of Mini-B had molecular interactions with DEPN-8 (Fig. 1, Table 2). This molecular biophysical behavior was consistent with the surface activity findings that 1.5% Mini-B increased the adsorption of DEPN-8 (Fig. 2), and enhanced its overall dynamic surface activity on the pulsating bubble (Fig. 3). Raising the content of Mini-B from 1.5% to 3% by weight relative to DEPN-8 did not lead to further increases in surface activity in captive bubble studies (Figs. 5, 6).

Although our current results show that DEPN-8+1.5% Mini-B has high overall surface activity, it is very likely that the lipid/peptide composition of synthetic exogenous surfactants can be optimized even further. Multiple chemical constituents interact to maximize surface activity in endogenous surfactant, and by analogy this is also true for related synthetic surfactants. In terms of lipid constituents, DEPN-8 and other disaturated PC analogs like SO_2_-lipid [35], [36] are designed with primary structural analogy to DPPC, the most prevalent single phospholipid in endogenous surfactant. However, endogenous surfactant also contains anionic components (phosphatidylglycerol, phosphatidylinositol, and phosphatidylserine) capable of interacting with positively charged amino acid residues in surfactant apoproteins. We have recently defined the synthesis of novel diether PG analogs (two phosphoglycerols and one phosphonoglycerol compound) for potential combination with DEPN-8 or SO_2_-lipid in synthetic exogenous lung surfactants [52]. These PG analogs are all structurally resistant to phospholipases A_1_ and A_2_, and the phosphonoglycerol is also resistant to phospholipase D. Initial surface activity assessments show that these PG analogs can increase the surface activity of DEPN-8 [52], and they are important candidates for further optimizing the lipid composition of synthetic surfactants containing DEPN-8 or SO_2_-lipid. In addition to modifying lipid headgroups, fatty chains can also be altered to include one or more double bonds as opposed to the 16∶0 moieties in DEPN-8. One of the foregoing diether PG analog compounds incorporates a 16∶1 chain to increase molecular fluidity in analogy with unsaturated glycerophospholipids in native surfactant [52].

In terms of optimizing the peptide composition of synthetic lung surfactants, the 34 amino acid Mini-B construct studied here retains important structural analogies to endogenous SP-B as noted earlier. However, this peptide does not incorporate all the molecular groups and interactions in the 79 amino acid primary sequence of human SP-B. Although DEPN-8+1.5% Mini-B had high dynamic surface activity and inhibition resistance to albumin (Figs. 3–6), maximum surface tension values were higher than those of CLSE during cycling on both the pulsating and captive bubble surfactometers (e.g., Figs. 5, 6). In addition, although DEPN-8+1.5% Mini-B had greatly increased adsorption compared to DEPN-8 alone (Fig. 2), the binary synthetic surfactant did not reach the same high level of adsorption achieved by CLSE (Fig. 2). Several modifications of Mini-B are being considered to further improve peptide activity, including focused amino acid substitutions or additions to increase molecular interactions with synthetic phospholipids and phosphonolipids. This includes specific changes in the primary sequence of Mini-B in the N- and C-terminal regions that, coupled with the addition of new synthetic lipids to DEPN-8, could significantly increase overall adsorption and dynamic surface activity in modified synthetic surfactants. Moreover, the synthesis of new SP-B-related peptides designed to form oligomers in analogy with native SP-B is also currently under active development, and synergy between SP-B peptides and novel SP-C/SP-A peptides in synthetic surfactants with lipid analogs is also being examined.

### Conclusions

This study documents that a fully-synthetic binary lung surfactant containing the diether phosphonolipid DEPN-8 combined with 1.5% (by weight) of the 34 amino acid Mini-B construct had very high overall dynamic surface activity on both the pulsating and captive bubble surfactometers. Mini-B interacted strongly at the molecular level with DEPN-8 based on plasmon resonance binding affinity studies and on FTIR analyses indicating that the peptide altered its relative content of α-helical and turn/bend conformation in DEPN-8 multilayers. DEPN-8 (0.5 mg/ml)+1.5% Mini-B had surface tension lowering ability similar to the active bovine surfactant extract CLSE (0.5 mg/ml) on the pulsating bubble, reaching minimum surface tensions of <1 mN/m at 10 min of bubble pulsation (20 cycles/min, 37°C, 50% area compression). DEPN-8 (2.5 mg/ml)+1.5% Mini-B and CLSE (2.5 mg/ml) also were comparable in reaching minimum surface tensions of <1 mN/m in the presence of serum albumin (3 mg/ml). Adsorbed films of DEPN-8+1.5% or 3% Mini-B and CLSE also were shown to reach low minimum surface tensions <1 mN/m during 10 successive cycles of quasi-static or dynamic compression on the captive bubble surfactometer. In addition, DEPN-8+1.5% Mini-B was chemically resistant to degradation by PLA_2_ *in vitro*, while CLSE was severely degraded by this enzyme. The high surface activity, albumin inhibition resistance, and phospholipase resistance of DEPN-8+1.5% Mini-B supports the continuing development of related fully-synthetic exogenous surfactants for treating inflammatory lung injuries such as ALI/ARDS.

## Materials and Methods

### (±)-trimethyl(3-phosphonopropyl)ammonium, mono(2,3-bis(hexadecyloxy)propyl) ester (DEPN-8)

DEPN-8 was synthesized and purified as described previously by Schwan, Notter, and co-workers [30], [36]. The chemical scheme for preparing DEPN-8 was based on the conversion of (±)-1-hexadecyloxy-2,3-propanediol to (±)-2,3-bis(hexadecyloxy)-1-propanol by way of hydroxyl protection at the 3-position, alkylation at the 2-hydroxyl group, and deprotection [30], [36]. Phosphonocholine placement involved treatment of (±)-2,3-bis(hexadecyloxy)-1-propanol with 3-bromopropylphosphono-di-chloridic acid prepared from 3-bromopropylphosphonic acid and PCl_5_ [30], followed by reaction with Me_3_N in CHCl_3_:MeOH:H_2_O (10∶10∶1). After concentration, the crude lipid was exposed to Amberlite® and subjected to flash chromatography with CHCl_3_:MeOH:H_2_O (60∶35∶5) as the elution solvent. Final purification of DEPN-8 was through recrystallization from CHCl_3_/acetone [30], as verified by both ^13^C and ^1^H NMR spectroscopy. In the former, only peaks expected for the product were observable, and ^1^H NMR exhibited a lone trimethylammonium resonance. DEPN-8 also gave a single spot on thin layer chromatography using a solvent system of 30∶9∶25∶7∶25 (by volume) chloroform:methanol:2-propanol:water:triethylamine (solvent system C of Touchstone et al [53]).

### Mini-B peptide

The 34 amino acid primary sequence of Mini-B is: NH_2 _- CWLCRALIKRIQAMIPKGGRMLPQLVCRLVLRCS - COOH [25]. Mini-B synthesis was done in a stepwise process starting with assembly as a linear sequence on an Applied Biosystems ABI 431A solid-phase peptide synthesizer configured for FastMoc™ chemistry [54]. A low substitution (0.3 mmole/gm) pre-derivatized Fmoc-serine (tBu) resin was used to minimize the formation of truncated sequences during synthesis, and all residues were double-coupled to the resin to insure optimal yield [25]. To facilitate the appropriate pairing of disulfide residues, cysteine residues at positions 1 and 33 were coupled using acid-labile Fmoc-Cys trityl [Fmoc-Cys(Trt)], and acid-resistant Fmoc-Cys acetamidomethyl (ACM) side chain-protecting groups were used for cysteine insertion at positions 4 and 27 [25]. Fmoc Gln(DMCP)-OH, which had greater solubility in coupling solvent [55], was used for the Glutamine residues as opposed to more conventional Fmoc-Gln(Trt)-OH. After synthesis of linear sequence, the crude peptide was cleaved from the resin and deprotected using a mixture of 0.75 gm phenol, 0.25 ml ethanedithiol, 0.5 ml of thioanisole, 0.5 ml of deionized water and 10 ml trifluoroacetic acid per gram of resin [25], [56]. The cleavage-deprotection mixture was chilled to 5°C and added to the resin, and then allowed to come to 25°C with continuous stirring over a period of 2 hrs to insure complete deprotection [25]. The crude peptide was removed by vacuum-assisted filtration, followed by washing the resin on a medium porosity sintered glass filter with trifluoroacetic acid and then dichloromethane to remove residual peptide. The filtrate was precipitated with ice cold tertiary butyl ether and separated by centrifugation at 2000×g for 10 min (several cycles of ether peptide precipitation and centrifugation were used to remove cleavage-deprotection byproducts). The crude peptide in the reduced state was dissolved in trifluoroethanol (TFE):10 mM HCl (1∶1, v∶v) and freeze-dried, followed by further purification using preparative scale HPLC [25]. The mass of final purified peptide was confirmed by MALDI TOF mass spectrometry, and peptide concentrations in physical studies were determined by UV absorbance at 280 nm [57].

### CLSE

CLSE was prepared by chloroform:methanol extraction of the large aggregate fraction of lung surfactant obtained by centrifugation (12,500×g for 30 min) of saline lavage from the intact lungs of freshly-killed calves as detailed previously [58]–[60].

### Phospholipase A_2_ (PLA_2_) and serum albumin for inhibition studies

PLA_2_ (Sigma Chemical, St. Louis, MO) was suspended in 0.15 M NaCl and 1.5 mM CaCl_2_ and incubated with surfactants dispersed in the same solvent for 30 min at 37°C (final enzyme concentration was 0.1 Units/ml) [3], [61]. Chemical degradation was assessed by determining phosphate levels [62] in thin layer chromatographic bands [53]. Albumin (Bovine serum, Fraction V, Sigma Chemical, 3 mg protein/ml) was combined with dispersed surfactants in 1.5M NaCl+1.5 mM CaCl_2_ and allowed to incubate at room temperature for 15–30 min prior to activity measurements on the pulsating bubble surfactometer.

### Lipid-Peptide Binding by Plasmon Resonance (Biacore)

Binding affinities of Mini-B for DEPN-8 and DPPC (Avanti Polar Lipids, Alabaster, AL) were measured with a Biacore 3000 system (Biacore, Uppsala, Sweden). Mini-B films were chemically linked to a CM5 sensor chip (BR-1000-14, research grade, containing a carboxymethylated dextran matrix covalently attached to a gold film) by ligand thiol-coupling of Cys 4 and Cys 27 in the peptide sequence. The chip surface was initially activated with 1∶1 EDC/NHS (EDC: 1-ethyl-3-(-3-dimethylaminopropyl)carbodiimide hydrochloride; NHS: N-hydroxysuccinimide), and the reactive disulfide groups were introduced using PDEA (2-(2-pyridinyldithio)ethaneamine hydrochloride). Mini-B was then introduced to the chip for the linkage reaction, which was subsequently deactivated by excess Cys/NaCl. Liposomes of DEPN-8 or of DPPC in running buffer (10 mM HEPES, 150 mM NaCl, 3 mM EDTA, 0.005% Surfactant P20, pH 7.4) were then flowed over the chip-linked peptide monolayer at a flow rate of 50 µl/min to determine binding affinity at 37°C. Binding associated with control medium containing no liposomes was subtracted from final affinity curves, and mean “on” and “off” rate constants (k_on_ and k_off_) and the dissociation equilibrium constant (KD = k_off_/k_on_) were calculated using BIAevaluation Software Version 4.1 based on curve fitting from measurements at six different lipid concentrations (0.1, 0.2, 0.3, 0.4, 0.5, and 0.6 µg/ml).

### FTIR and CD spectroscopy

Infrared spectra were recorded at 25°C using a Bruker Vector 22™ FTIR spectrometer (Pike Technologies) with a DTGS detector, averaged over 256 scans at a gain of 4 and a resolution of 2 cm^−1^. Lipid and peptide samples were initially freeze-dried several times from 10 mM HCl to remove any interfering counter ions. Films of DEPN-8 or DEPN-8:Mini-B (10∶1 mole∶mole) for FTIR were prepared by air-drying from chloroform:TFE (1∶1, v∶v) onto a 50×20×2 mm, 45 degree ATR crystal [27], [51], and hydrated by passing deuterium-saturated nitrogen gas through the sample chamber for one hour prior to spectroscopy. Films of Mini-B alone were air-dried from TFE onto the ATR crystal surface, and then carefully overlaid with TFE to insure solvent saturation of the peptide. Proportions of α-helix, turn/bend, β-sheet, and disordered conformations were determined by Fourier self-deconvolutions for band narrowing and area calculations of component peaks of the FTIR spectra using curve-fitting software supplied by Galactic Software (GRAMS/32, version 5; Galactic Industries Corp., Salem, NH). The FTIR frequency limits used for the different structures were: α-helix (1662–1645 cm^−1^), β-sheet (1637–1613 and 1710–1682 cm^−1^), turn/bend (1682–1662 cm^−1^), and disordered or random (1650–1637 cm^−1^) [63]. CD spectra (185–260 nm) were also made for Mini-B in 4∶6 v∶v TFE:10 mM phosphate buffer (pH 7.4) using a JASCO 715 spectropolarimeter (Jasco Inc., Easton, MD) fitted with a thermoelectric temperature controller and calibrated for wavelength and optical rotation using (+)-10-camphorsulphonic acid [64]. Peptide samples in 0.01 cm pathlength cells were scanned at a rate of 20 nm/min (sample interval 0.2 nm) at 25°C. CD spectra for Mini-B were baseline-corrected by subtracting spectra for control peptide-free solutions, and absorbance was expressed as mean residue ellipticity (MRE). Quantitative estimates of the secondary structural contributions from CD spectra were made with SELCON 3 [28] using the spectral basis set for membrane proteins implemented in the Olis Global Works™ software package (Olis Inc., Bogart, GA).

### Adsorption apparatus

Adsorption experiments were done at 37±0.5°C in a Teflon® dish with a 35 ml subphase (0.15 M NaCl+1.5 mM CaCl_2_ ) stirred to minimize diffusion resistance as described previously [65], [66]. At time zero, a bolus of surfactant containing 2.5 mg lipid in 5 ml of 0.15 M NaCl+1.5 mM CaCl_2_ was injected into the stirred subphase, and adsorption surface pressure (surface tension lowering below that of the pure subphase) was measured as a function of time by the force on a partially submerged, sandblasted platinum Wilhelmy slide [65], [66]. The final surfactant concentration for adsorption studies was uniform at 0.0625 mg phospholipid/ml (2.5 mg surfactant phospholipid/40 ml of final subphase).

### Pulsating bubble surfactometer methods

The pulsating bubble surfactometer (General Transco, Largo, FL; formerly Electronetics Corporation, Amherst, NY) used in activity studies was based on the original design of Enhorning [67]. Surfactant preparations (DEPN-8+1.5% Mini-B or CLSE) were dissolved in chloroform, dried under nitrogen, and dispersed in either 0.15 M NaCl+1.5 mM CaCl_2_ or 0.15 M NaCl. Dispersion was by probe sonication on ice with 3–4 bursts of 15 sec duration each (W220F Sonicator, 40 watts power). A 40 µl volume of dispersed surfactant was added to a plastic sample holder mounted on the pulsator unit of the bubble surfactometer. A small air bubble was then formed and pulsated at a physiological rate of 20 cycles/min between maximum and minimum radii of 0.55 and 0.4 mm (50% surface area compression for a truncated sphere) [67]. The pressure in the liquid phase was measured with a precision transducer, and surface tensions at minimum and maximum bubble radius (minimum and maximum surface tensions) were calculated as a function of time of pulsation from the measured pressure drop across the air-water interface using the Laplace equation for a sphere [67], [68]. Surfactant concentration was 1.0 or 2.5 mg phosphonolipid/ml. Measurements were made at 37±0.5°C.

### Captive bubble surfactometer

The captive bubble instrument used was a fully computerized version of that described in detail elsewhere [42]–[44]. In brief, the sample chamber of the apparatus was cut from high-quality cylindrical glass tubing (10 mm inner diameter). A Teflon® piston with a tight O-ring seal was fitted into the glass tubing from the top end, with a plug of buffered 1% agarose gel inserted between the piston and the surfactant solution that was added through a stainless steel port from the other end of the sample chamber. The chamber and piston were vertically mounted in a steel rack, the height of which was regulated by a precision micrometer gear. In a typical experiment, the chamber was filled with a buffered salt solution (140 mM NaCl, 10 mM HEPES, 2.5 mM CaCl_2_, pH 6.9) containing 10% sucrose. One µl of surfactant solution containing 35 µg of lipid was added to this subphase, which was stirred by a small magnetic bar at 37°C. The subphase volume in the sample chamber averaged 0.7 ml (0.5–1 ml), resulting in a final average surfactant lipid concentration of 50 µg/ml (35–75 µg/ml). An air bubble approximately 7 mm in diameter (∼200 µl in volume) was then introduced within the sample chamber and subjected to cyclic volume (surface area) changes by systematically varying the height of the steel rack following a 5 min pause to allow adsorption to the air-water interface. The ionic composition of the buffered agarose plug minimized bubble adhesion to the plug during cycling, so that an uninterrupted bubble interface was maintained. Surface studies utilized a compression ratio of approximately 5∶1 (maximum area/minimum area) and two sets of cycling conditions: (1) initial quasi-static compression/expansion (10 cycles over 90 min including a 2 min pause between each cycle) followed by 10 cycles of dynamic compression/expansion (20 cycles/min). During quasi-static cycling, bubble size was varied in a stepwise fashion involving a 3-s change in volume followed by a 4-s delay while the film was allowed to “relax”. Compression cycles were halted when bubble height no longer decreased as bubble volume was decreased. In dynamic cycling studies, bubble size was smoothly varied over the same size range as in the quasi-static studies. Bubble images were continuously monitored during compression-decompression using a digital video camera (PULNIX Model TM-200, Pulmix America Inc, Sunnyvale, CA) and a professional video recorder (Panasonic AG-1980P, Secaucus, NJ 07094) coupled to a computer with an Intel Pentium 4 processor. Selected single frames stored in RAM were subsequently subjected to image processing and analysis [69]. Bubble areas and volumes were calculated by an original algorithm relating bubble height and diameter to areas of revolution, and bubble surface tension was determined by the method of Malcolm and Elliot [70].
